# Commentary: Vagal Tank Theory: The Three Rs of Cardiac Vagal Control Functioning – Resting, Reactivity, and Recovery

**DOI:** 10.3389/fnins.2019.01300

**Published:** 2019-12-05

**Authors:** Laura Hottenrott, Sascha Ketelhut, Kuno Hottenrott

**Affiliations:** ^1^Faculty of Sport Science, Ruhr-University, Bochum, Germany; ^2^Department of Training Science and Sports Medicine, Institute of Sports Science, Martin-Luther-University Halle-Wittenberg, Halle, Germany

**Keywords:** orthostatic test, cardiac vagal activity, endurance training, self-regulation, vagal tone, autonomic control, heart rate variability, parasymapthetic nervous system

The authors of this paper (Laborde et al., 2018) developed a new theory, they call “Vagal Tank Theory,” which helps to better understand the complexity of self-regulatory interactions and their mechanisms, especially from a neurobiological and psychological point of view. To operationalize their theoretical assumptions, they analyze the activity of the parasympathetic nervous system by measuring the beat-to-beat variation of the heart rate and calculating vagal parameters of heart rate variability (HRV) from time and frequency domain analysis.

Laborde and colleagues provide a very promising approach for investigating factors that positively and negatively influence cardiac vagal activity by self-regulatory mechanisms. The authors propose that cardiac vagal control has a barometer role reflecting how efficiently self-regulatory resources are mobilized. Therefore, they use the metaphor of the vagal tank which can be depleted and replenished according to different situations and tasks. For the cardiac vagal control analysis, the authors suggest three different levels (Resting, Reactivity, Recovery) as they all represent different levels of adaptability. Their theory is based on the assumption that the filling state of the vagal tank significantly reflects the self-regulation ability in the three levels.

Level 1 (Resting) refers to a baseline level and is measured in a sitting, standing or supine position depending on the research question. Laborde et al. assume that better self-regulation is accompanied by a higher vagal activity at rest (“The higher the better”). According to the authors, a fuller tank is related to a better management function (decision-making, working memory, rationality, etc.), better stress management, better emotional regulation, and better health in general.

Level 2 (Reactivity) describes the change from baseline caused by a specific event or stressor, which may be cognitive, emotional, or physical in nature. The intervention or stressor may lead to depletion or replenishment of the tank (see Figures 3, 4 in Laborde et al., 2018). The authors state, that the reactivity, especially the patterns of change in cardiac vagal activity reflects the effectiveness of the self-regulation mechanisms.

The transition from level 2 to level 3 (Recovery) is described as a process of restoration to the initial baseline level (B) or a new higher or lower level (A or C). Based on these measurements conclusions about vagal recovery (vagal rebound) can be drawn.

Even though the authors conceived a very comprehensive theory this commentary tries to further develop the authors' model for the application in sports and exercise. Which factors may contribute to the filling of the vagus tank above the initial level after elimination of the depleting or replenishing factors does not clearly emerge from the vagal tank model (Figures 3, 4, dotted line A in Laborde et al., 2018), although there are settings/conditions that can promote a regenerative state of the organism above the level of the initial resting phase (e.g., during sleep phases or with predetermined slow deep breathing).

Laborde et al. expect the “Vagal Tank Theory” to be transferable and applicable to various areas including medicine, school, work, sport, and daily life. However, one must emphasize, that the model only applies to single events and is not applicable when it comes to a series of events. This is particularly relevant in relation to physical activity. Even though cardiac vagal activity may drop during an acute bout of exercise, regular physical activity (aerobic training) has been shown to increase resting cardiac vagal activity (De Meersman, 1993; Stanley et al., 2013; Bellenger et al., 2016a).

The authors' thesis “the higher the better” related to resting cardiac vagal activity can be problematic in the context of organismic changes and adaptations and often does not apply to physical activity. In endurance sports, for example, a very high resting cardiac vagal activity can lead to reductions in performance. In this state of non-functional overreaching, not only physical performance but also motivational and emotional regulation can be limited (Meeusen et al., 2013). In some cases, strenuous exercise can even induce cardiovascular sympathetic overactivity (Dalla Vecchia et al., 2019).

Furthermore, the model (Figure 3 in Laborde et al., 2018) doesn't explain which changes occur when there is already a saturation effect of vagal activity, e.g., in well-trained endurance athletes (Buchheit et al., 2004). In this case additional training interventions may not cause a further increase in vagal activity in a resting supine or sitting measurement (Plews et al., 2013; Bellenger et al., 2016a,b; Hottenrott and Hoos, 2017). To detect further changes in this saturated state the model shouldn't be limited to the analysis of cardiac vagal activity in just one body position but should consider assessing cardiac vagal activity in two different body positions, to better index adaptation mechanisms (e.g., supine and standing) and thus make the “Vagal Tank Theory” more applicable to endurance sports. In this regard, the orthostatic test has proved its worth (Le Meur et al., 2013; Bellenger et al., 2016b). When applying the findings of the orthostatic test summarized in the model from Hottenrott and Hoos (2017) to the original “Vagal Tank Theory” from Laborde et al. we developed the following modified model (Figure 1).

**Figure 1:**
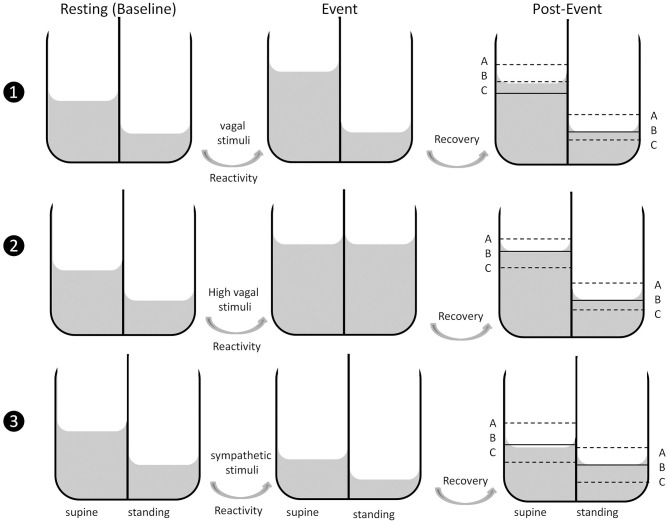
Adjusted vagal tank model according to Laborde et al. (2018) for application in sports including the orthostatic test. Vagal tank with vagal stimuli (1), with high vagal stimuli (2), and sympathetic stimuli (3). In regards to the post-event: A, B, and C display potential levels of cardiac vagal control at post-event after recovery.

Three typical responses to training stimuli which have been shown in several studies are displayed. Situation 1 shows the response to an aerobic endurance training lasting several days up to 2–3 weeks, which represents a moderate parasympathetic stimuli (Aubert et al., 2003; Le Meur et al., 2013, Plews et al., 2013; Stanley et al., 2013; Bellenger et al., 2016a). Situation 2 shows the response to an increase in the aerobic training volume for several days up to 2–3 weeks by 100–200% from the initial training load (at baseline), portraying a very high parasympathetic stimulus (Bellenger et al., 2016a,b; Hottenrott and Hoos, 2017). Situation 3 shows the reaction to several days of high-intensity interval training (HIIT) or a micro-shock-cycle, triggering a sympathetic reaction/displaying a sympathetic stimulus (Hottenrott and Hoos, 2017; Schneider et al., 2018, 2019).

For the determination of the baseline level, no excessive training should take place, but rather regenerative training, as this would affect the baseline level. High-intensity training can reduce resting cardiac vagal activity and a high volume of aerobic training can increase resting cardiac vagal activity (Buchheit and Gindre, 2006).

We compliment Laborde et al. on their theory and their model. The Vagal Tank Theory with the three R's is a promising tool to demonstrate and visualize changes in cardiac vagal activity. However, an extension of the model with testing in two body-positions would be very helpful for the application in sports. Physical exercise has already been mentioned by Laborde et al. as a field of application, without giving specific answers or practical recommendations for the implementation. This extended model, which includes the orthostatic test, provides Laborde et al. original model with an additional field of application.

In summary, the purpose of this commentary was to further develop the “Vagal Tank Theory” by Laborde and colleagues to provide a practical model that can be integrated into future research and is applicable for coaches and athletes in the field. With the use of the orthostatic test, we offer a non-invasive possibility to detect and evaluate the transition from functional overreaching (FOR) to overtraining (OT) in elite endurance athletes. A regular and standardized application of the orthostatic test of e.g., 2 min supine followed by 2 min standing measurement in the morning after awaking (Schäfer et al., 2015) can serve as an easily obtainable “tool” for athletes and coaches to operationalize training load.

## Author Contributions

LH and KH designed the model and wrote the first draft. SK provided critical comments and contributed to the final manuscript.

### Conflict of Interest

The authors declare that the research was conducted in the absence of any commercial or financial relationships that could be construed as a potential conflict of interest.
